# Bis(dicyclo­hexyl­aminium) 2-carb­oxy­methyl-2-hy­droxy­succinate ethanol monosolvate

**DOI:** 10.1107/S1600536812035684

**Published:** 2012-08-25

**Authors:** Mahsa Foroughian, Behrouz Notash, Abbas Shafiee, Hossein Aghabozorg, Alireza Foroumadi

**Affiliations:** a Faculty of Chemistry, Islamic Azad University, North Tehran Branch, Tehran, Iran; b Department of Chemistry, Shahid Beheshti University, G. C., Evin, Tehran 1983963113, Iran; c Drug Design & Development Research Center, Tehran, University of Medical Sciences, Tehran, Iran; d Neuroscience Research Center, Kerman University of Medical Sciences, Kerman, Iran

## Abstract

In the title compound, 2C_12_H_24_N^+^·C_6_H_6_O_7_^2−^·C_2_H_6_O, the cyclo­hexane rings of the cations adopt chair conformations. In the anion, intra­molecular O—H⋯O hydrogen bonds occur. In the crystal, the cations link with the anions *via* N—H⋯O hydrogen bonds. Weak C—H⋯O hydrogen bonds are also observed. The hy­droxy group of the ethanol solvent mol­ecule is disordered over two sets of sites with an occupancy ratio of 0.766 (5):0.234 (5).

## Related literature

For background to proton-transfer compounds, see: Aghabozorg *et al.* (2008 ▶). For related structures, see: Aghabozorg *et al.* (2011*a* ▶,*b* ▶,*c* ▶); Foroughian *et al.* (2011 ▶); Sharif *et al.* (2010 ▶). For similar proton-transfer structures, see: Jin *et al.* (2004 ▶); Chen *et al.* (2003 ▶).
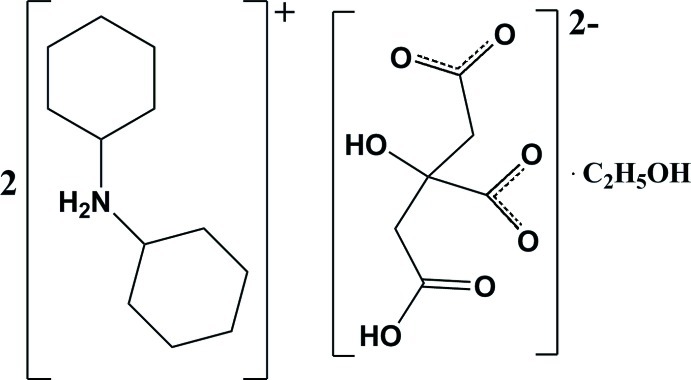


## Experimental

### 

#### Crystal data

2C_12_H_24_N^+^·C_6_H_6_O_7_^2−^·C_2_H_6_O
*M* *_r_* = 600.82
Triclinic, 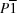

*a* = 10.054 (2) Å
*b* = 12.329 (3) Å
*c* = 13.908 (3) Å
α = 99.77 (3)°
β = 92.17 (3)°
γ = 95.98 (3)°
*V* = 1687.0 (7) Å^3^
*Z* = 2
Mo *K*α radiation
μ = 0.08 mm^−1^
*T* = 120 K
0.34 × 0.32 × 0.30 mm

#### Data collection

Stoe IPDS 2T diffractometer
18454 measured reflections
9009 independent reflections
6897 reflections with *I* > 2σ(*I*)
*R*_int_ = 0.046

#### Refinement

*R*[*F* ^2^ > 2σ(*F* ^2^)] = 0.063
*wR*(*F* ^2^) = 0.136
*S* = 1.09
9009 reflections
420 parameters
1 restraint
H atoms treated by a mixture of independent and constrained refinement
Δρ_max_ = 0.36 e Å^−3^
Δρ_min_ = −0.37 e Å^−3^

### 

Data collection: *X-AREA* (Stoe & Cie, 2005 ▶); cell refinement: *X-AREA*; data reduction: *X-RED* (Stoe & Cie, 2005 ▶); program(s) used to solve structure: *SHELXS97* (Sheldrick, 2008 ▶); program(s) used to refine structure: *SHELXL97* (Sheldrick, 2008 ▶); molecular graphics: *ORTEP-3 for Windows* (Farrugia, 1997 ▶); software used to prepare material for publication: *WinGX* (Farrugia, 1999 ▶).

## Supplementary Material

Crystal structure: contains datablock(s) I, global. DOI: 10.1107/S1600536812035684/xu5604sup1.cif

Structure factors: contains datablock(s) I. DOI: 10.1107/S1600536812035684/xu5604Isup2.hkl

Supplementary material file. DOI: 10.1107/S1600536812035684/xu5604Isup3.cml

Additional supplementary materials:  crystallographic information; 3D view; checkCIF report

## Figures and Tables

**Table 1:** Hydrogen-bond geometry (Å, °)

*D*—H⋯*A*	*D*—H	H⋯*A*	*D*⋯*A*	*D*—H⋯*A*
N1—H1*A*⋯O4^i^	0.93 (2)	1.84 (2)	2.7651 (19)	170 (2)
N1—H1*B*⋯O7	0.87 (2)	2.31 (2)	3.033 (2)	140.8 (19)
N1—H1*B*⋯O4	0.87 (2)	2.07 (2)	2.8390 (19)	146 (2)
N2—H2*C*⋯O6^ii^	0.90 (2)	1.90 (2)	2.794 (2)	170.3 (19)
N2—H2*D*⋯O5	0.93 (2)	1.86 (2)	2.751 (2)	160 (2)
O2—H2⋯O3	0.94 (3)	1.56 (3)	2.499 (2)	174 (3)
O7—H5⋯O5	0.86 (3)	1.92 (3)	2.6678 (19)	145 (2)
C18—H18*A*⋯O2^iii^	0.99	2.51	3.405 (2)	150
C20—H20*A*⋯O1^iii^	0.99	2.47	3.457 (2)	172
C32—H32*A*⋯O8*A* ^iv^	0.99	2.56	3.458 (4)	151

Symmetry codes: (i) 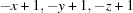; (ii) 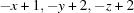; (iii) 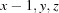; (iv) 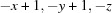.

**Fig. 1.**
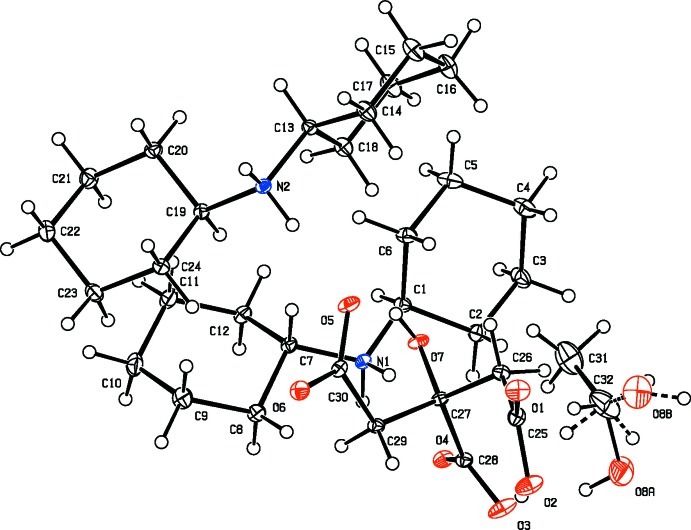
The molecular structure of the title compound. Displacement ellipsoids are drawn at 30% probability level.

**Fig. 2.**
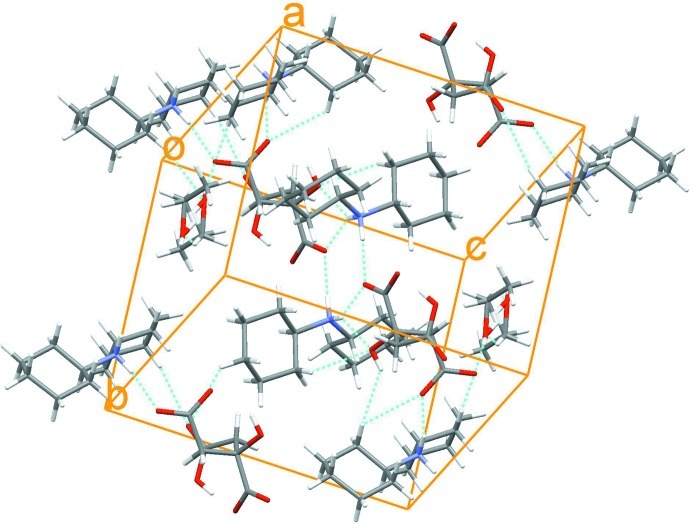
The packing diagram of the title compound showing intermolecular hydrogen bonding as blue dash lines.

## supplementary crystallographic information

### Comment

Proton transfer compounds are important in chemistry, biochemistry and medicinal chemistry. Our research group focus on synthesis of new proton transfer compounds especially from pyridine dicarboxcylic acids (Aghabozorg *et al.* 2008) and different organic bases with nitrogen donor sites such as propane-1,3-diamine (Aghabozorg *et al.*, 2011*a*), diethylenetriamine (Aghabozorg *et al.*, 2011*c*), 2-amino-4-methylpyridine (Aghabozorg *et al.*, 2011**b*;* Sharif *et al.*, 2010) and 2,3-diaminopyridine (Foroughian *et al.*, 2011). There are also several proton transfer compound have been reported in which citrate exist as anion (Chen *et al.*, 2003) and dicyclohexylamine as cation (Jin *et al.*, 2004).

The asymmetric unit of the title compound consist of two protonated cyclohexylamine as cation, one deprotonated citrate as anion, and one ethanol molecule. The asymmetric unit of the title compound is shown in Fig.1. In the crystal structure of the title compound, there are extensive O—H···O, N—H···O and weak intermolecular C—H···O hydrogen bonds. These hydrogen bonds play important role in the stabilization of crystal packing of the title compound (Table 1 & Fig. 2).

### Experimental

The solution of citric acid monohydrate (0.334 g, 1 mmol) in 5 ml ethanol was added to solution of dicyclohexylamine (0.6 ml, 3 mmol) in 10 ml ethanol in 1:3 molar ratios. The reaction mixture was stirred for 3 h at 298 K. The colorless crystals of the title compound appeared after slow evaporation of solvent at room temperature in darkness.

### Refinement

The hydrogen atoms bonded to O and N atoms were found in difference Fourier map and refined isotropically. The hydroxyl hydrogen atom (H8BB) was refined with *U*iso(H) = 1.2 *U*eq(O) and distance restraints of O—H 0.89 (2) and also C32—H8BB = 1.93 (4) Å. The C—H protons were positioned geometrically and refined as riding atoms with C—H = 0.99 Å and *U*iso(H) = 1.2 *U*eq(C) for CH_2_ groups, C—H = 0.98 Å and *U*iso(H) = 1.5 *Ueq*(C) for methyl group. Hydroxyl group of ethanol solvent molecule was disordered over two sites with relative occupancies of 0.766 (5) and 0.234 (5).

### Crystal data

**Table:**

2C_12_H_24_N^+^·C_6_H_6_O_7_^2^^−^·C_2_H_6_O	*Z* = 2
*M**_r_* = 600.82	*F*(000) = 660
Triclinic, *P*1	*D*_x_ = 1.183 Mg m^−^^3^
Hall symbol: -P 1	Mo *K*α radiation, λ = 0.71073 Å
*a* = 10.054 (2) Å	Cell parameters from 9009 reflections
*b* = 12.329 (3) Å	θ = 2.4–29.1°
*c* = 13.908 (3) Å	µ = 0.08 mm^−^^1^
α = 99.77 (3)°	*T* = 120 K
β = 92.17 (3)°	Block, colorless
γ = 95.98 (3)°	0.34 × 0.32 × 0.30 mm
*V* = 1687.0 (7) Å^3^	

### Data collection

**Table:**

Stoe IPDS 2T diffractometer	6897 reflections with *I* > 2σ(*I*)
Radiation source: fine-focus sealed tube	*R*_int_ = 0.046
Graphite monochromator	θ_max_ = 29.1°, θ_min_ = 2.4°
rotation method scans	*h* = −13→12
18454 measured reflections	*k* = −16→16
9009 independent reflections	*l* = −19→19

### Refinement

**Table:**

Refinement on *F*^2^	Primary atom site location: structure-invariant direct methods
Least-squares matrix: full	Secondary atom site location: difference Fourier map
*R*[*F*^2^ > 2σ(*F*^2^)] = 0.063	Hydrogen site location: inferred from neighbouring sites
*wR*(*F*^2^) = 0.136	H atoms treated by a mixture of independent and constrained refinement
*S* = 1.09	*w* = 1/[σ^2^(*F*_o_^2^) + (0.0469*P*)^2^ + 0.9825*P*] where *P* = (*F*_o_^2^ + 2*F*_c_^2^)/3
9009 reflections	(Δ/σ)_max_ < 0.001
420 parameters	Δρ_max_ = 0.36 e Å^−^^3^
1 restraint	Δρ_min_ = −0.37 e Å^−^^3^

### Special details

**Table:**

Geometry. All e.s.d.'s (except the e.s.d. in the dihedral angle between two l.s. planes) are estimated using the full covariance matrix. The cell e.s.d.'s are taken into account individually in the estimation of e.s.d.'s in distances, angles and torsion angles; correlations between e.s.d.'s in cell parameters are only used when they are defined by crystal symmetry. An approximate (isotropic) treatment of cell e.s.d.'s is used for estimating e.s.d.'s involving l.s. planes.
Refinement. Refinement of *F*^2^ against ALL reflections. The weighted *R*-factor *wR* and goodness of fit *S* are based on *F*^2^, conventional *R*-factors *R* are based on *F*, with *F* set to zero for negative *F*^2^. The threshold expression of *F*^2^ > σ(*F*^2^) is used only for calculating *R*-factors(gt) *etc*. and is not relevant to the choice of reflections for refinement. *R*-factors based on *F*^2^ are statistically about twice as large as those based on *F*, and *R*- factors based on ALL data will be even larger.

### Fractional atomic coordinates and isotropic or equivalent isotropic displacement parameters (Å^2^)

**Table:**

	*x*	*y*	*z*	*U*_iso_*/*U*_eq_	Occ. (<1)
O1	0.92627 (13)	0.99218 (11)	0.75222 (10)	0.0263 (3)	
O2	0.96806 (13)	0.82764 (12)	0.67635 (11)	0.0297 (3)	
O3	0.82351 (13)	0.66771 (12)	0.57673 (11)	0.0303 (3)	
O4	0.60847 (12)	0.60824 (10)	0.54461 (9)	0.0188 (2)	
O5	0.54560 (12)	0.90464 (11)	0.81394 (9)	0.0226 (3)	
O6	0.66502 (13)	0.84223 (11)	0.92729 (9)	0.0218 (3)	
O7	0.52666 (11)	0.79006 (10)	0.63220 (9)	0.0171 (2)	
N1	0.32477 (13)	0.59787 (11)	0.53775 (10)	0.0137 (3)	
N2	0.33629 (13)	1.00067 (11)	0.90248 (10)	0.0136 (3)	
C1	0.25681 (16)	0.64627 (13)	0.45948 (11)	0.0154 (3)	
H1	0.1692	0.6005	0.4385	0.019*	
C2	0.34569 (18)	0.63827 (15)	0.37245 (12)	0.0210 (3)	
H2A	0.3576	0.5597	0.3488	0.025*	
H2B	0.4351	0.6790	0.3933	0.025*	
C3	0.2835 (2)	0.68686 (16)	0.28926 (13)	0.0263 (4)	
H3A	0.3456	0.6853	0.2356	0.032*	
H3B	0.1990	0.6408	0.2633	0.032*	
C4	0.2548 (2)	0.80575 (16)	0.32435 (14)	0.0290 (4)	
H4A	0.2097	0.8335	0.2704	0.035*	
H4B	0.3402	0.8535	0.3438	0.035*	
C5	0.1658 (2)	0.81167 (16)	0.41111 (14)	0.0271 (4)	
H5A	0.0777	0.7686	0.3901	0.033*	
H5B	0.1506	0.8897	0.4341	0.033*	
C6	0.22998 (18)	0.76555 (14)	0.49538 (12)	0.0202 (3)	
H6A	0.3151	0.8115	0.5197	0.024*	
H6B	0.1692	0.7680	0.5499	0.024*	
C7	0.25759 (16)	0.59934 (13)	0.63255 (11)	0.0150 (3)	
H7	0.2641	0.6776	0.6678	0.018*	
C8	0.33355 (17)	0.53250 (15)	0.69423 (12)	0.0191 (3)	
H8B	0.4291	0.5630	0.7029	0.023*	
H8C	0.3280	0.4546	0.6604	0.023*	
C9	0.27399 (19)	0.53693 (17)	0.79390 (13)	0.0264 (4)	
H9A	0.2874	0.6141	0.8299	0.032*	
H9B	0.3213	0.4904	0.8322	0.032*	
C10	0.1245 (2)	0.49606 (19)	0.78365 (14)	0.0297 (4)	
H10A	0.1119	0.4160	0.7559	0.036*	
H10B	0.0872	0.5061	0.8490	0.036*	
C11	0.04926 (18)	0.55885 (17)	0.71777 (13)	0.0240 (4)	
H11A	0.0524	0.6373	0.7496	0.029*	
H11B	−0.0458	0.5269	0.7087	0.029*	
C12	0.10981 (16)	0.55305 (14)	0.61780 (12)	0.0180 (3)	
H12A	0.1011	0.4753	0.5834	0.022*	
H12B	0.0614	0.5968	0.5774	0.022*	
C13	0.28519 (16)	1.06479 (13)	0.82796 (11)	0.0153 (3)	
H13	0.1982	1.0910	0.8488	0.018*	
C14	0.38706 (19)	1.16572 (14)	0.82771 (13)	0.0219 (3)	
H14A	0.4745	1.1412	0.8086	0.026*	
H14B	0.3997	1.2107	0.8942	0.026*	
C15	0.3375 (2)	1.23593 (16)	0.75567 (14)	0.0282 (4)	
H15A	0.2540	1.2654	0.7783	0.034*	
H15B	0.4055	1.2996	0.7541	0.034*	
C16	0.3108 (2)	1.16857 (17)	0.65307 (14)	0.0289 (4)	
H16A	0.3962	1.1466	0.6271	0.035*	
H16B	0.2727	1.2146	0.6095	0.035*	
C17	0.2137 (2)	1.06527 (16)	0.65413 (14)	0.0265 (4)	
H17A	0.2030	1.0201	0.5877	0.032*	
H17B	0.1249	1.0876	0.6722	0.032*	
C18	0.26265 (17)	0.99487 (14)	0.72644 (12)	0.0189 (3)	
H18A	0.1952	0.9307	0.7277	0.023*	
H18B	0.3473	0.9664	0.7053	0.023*	
C19	0.25200 (15)	0.89738 (13)	0.91880 (11)	0.0144 (3)	
H19	0.2483	0.8401	0.8582	0.017*	
C20	0.10990 (16)	0.92061 (14)	0.94244 (13)	0.0182 (3)	
H20A	0.0655	0.9447	0.8862	0.022*	
H20B	0.1126	0.9811	0.9995	0.022*	
C21	0.03013 (18)	0.81608 (15)	0.96495 (13)	0.0223 (3)	
H21A	−0.0620	0.8319	0.9804	0.027*	
H21B	0.0240	0.7569	0.9066	0.027*	
C22	0.09666 (18)	0.77620 (15)	1.05125 (13)	0.0222 (3)	
H22A	0.0447	0.7076	1.0634	0.027*	
H22B	0.0974	0.8332	1.1107	0.027*	
C23	0.24032 (18)	0.75376 (14)	1.02973 (13)	0.0217 (3)	
H23A	0.2385	0.6903	0.9753	0.026*	
H23B	0.2842	0.7335	1.0881	0.026*	
C24	0.32186 (16)	0.85492 (14)	1.00240 (12)	0.0178 (3)	
H24A	0.3356	0.9147	1.0603	0.021*	
H24B	0.4110	0.8350	0.9827	0.021*	
C25	0.88918 (16)	0.90695 (14)	0.69607 (12)	0.0190 (3)	
C26	0.75014 (16)	0.88667 (13)	0.64500 (12)	0.0164 (3)	
H26A	0.7601	0.8859	0.5744	0.020*	
H26B	0.7008	0.9502	0.6697	0.020*	
C27	0.66314 (15)	0.77906 (13)	0.65679 (11)	0.0130 (3)	
C28	0.70082 (16)	0.67714 (13)	0.58628 (11)	0.0149 (3)	
C29	0.67698 (16)	0.75552 (13)	0.76197 (11)	0.0149 (3)	
H29A	0.6271	0.6825	0.7641	0.018*	
H29B	0.7726	0.7505	0.7783	0.018*	
C30	0.62628 (15)	0.84224 (13)	0.84065 (12)	0.0153 (3)	
C31	0.2132 (3)	0.4472 (2)	0.0602 (2)	0.0539 (7)	
H31A	0.1819	0.3679	0.0455	0.081*	
H31B	0.1890	0.4820	0.0047	0.081*	
H31C	0.1712	0.4815	0.1184	0.081*	
C32	0.3590 (3)	0.4624 (2)	0.0782 (2)	0.0530 (7)	
H32A	0.3951	0.5274	0.0504	0.064*	0.766 (5)
H32B	0.3792	0.4802	0.1497	0.064*	0.766 (5)
H32C	0.3758	0.4291	0.1360	0.064*	0.234 (5)
H32D	0.3944	0.4132	0.0246	0.064*	0.234 (5)
H2C	0.344 (2)	1.0483 (18)	0.9596 (16)	0.019 (5)*	
H1A	0.337 (2)	0.5254 (19)	0.5112 (16)	0.023 (5)*	
H2D	0.419 (2)	0.9799 (19)	0.8832 (16)	0.025 (6)*	
H1B	0.407 (2)	0.6300 (19)	0.5486 (16)	0.024 (5)*	
H5	0.503 (2)	0.835 (2)	0.6806 (19)	0.035 (6)*	
H2	0.919 (3)	0.767 (3)	0.636 (2)	0.065 (9)*	
O8A	0.4261 (3)	0.3748 (2)	0.0413 (2)	0.0558 (8)	0.766 (5)
O8B	0.4416 (8)	0.5459 (7)	0.1016 (6)	0.049 (2)	0.234 (5)
H8AA	0.383 (4)	0.312 (4)	0.052 (3)	0.068 (13)*	0.766 (5)
H8BB	0.502 (12)	0.568 (13)	0.062 (9)	0.082*	0.234 (5)

### Atomic displacement parameters (Å^2^)

**Table:**

	*U*^11^	*U*^22^	*U*^33^	*U*^12^	*U*^13^	*U*^23^
O1	0.0248 (6)	0.0215 (6)	0.0289 (7)	−0.0006 (5)	−0.0063 (5)	−0.0028 (5)
O2	0.0155 (6)	0.0259 (7)	0.0420 (8)	0.0025 (5)	−0.0027 (5)	−0.0095 (6)
O3	0.0170 (6)	0.0287 (7)	0.0384 (8)	0.0066 (5)	0.0003 (5)	−0.0154 (6)
O4	0.0185 (6)	0.0143 (5)	0.0208 (6)	0.0015 (4)	0.0002 (4)	−0.0041 (4)
O5	0.0212 (6)	0.0278 (7)	0.0190 (6)	0.0126 (5)	0.0017 (5)	−0.0014 (5)
O6	0.0260 (6)	0.0249 (6)	0.0139 (5)	0.0063 (5)	0.0013 (5)	−0.0001 (5)
O7	0.0118 (5)	0.0188 (6)	0.0191 (6)	0.0052 (4)	−0.0030 (4)	−0.0027 (5)
N1	0.0129 (6)	0.0136 (6)	0.0140 (6)	0.0023 (5)	0.0007 (5)	0.0003 (5)
N2	0.0138 (6)	0.0144 (6)	0.0125 (6)	0.0030 (5)	0.0006 (5)	0.0013 (5)
C1	0.0171 (7)	0.0152 (7)	0.0141 (7)	0.0033 (6)	−0.0010 (6)	0.0024 (6)
C2	0.0274 (9)	0.0209 (8)	0.0168 (8)	0.0079 (7)	0.0054 (6)	0.0050 (6)
C3	0.0408 (11)	0.0239 (9)	0.0162 (8)	0.0078 (8)	0.0019 (7)	0.0067 (7)
C4	0.0412 (11)	0.0228 (9)	0.0249 (9)	0.0084 (8)	−0.0038 (8)	0.0083 (7)
C5	0.0334 (10)	0.0212 (9)	0.0275 (9)	0.0119 (7)	−0.0066 (8)	0.0027 (7)
C6	0.0249 (8)	0.0181 (8)	0.0179 (8)	0.0088 (6)	−0.0015 (6)	0.0005 (6)
C7	0.0164 (7)	0.0168 (7)	0.0113 (7)	0.0030 (6)	0.0010 (5)	−0.0001 (5)
C8	0.0186 (8)	0.0236 (8)	0.0155 (7)	0.0046 (6)	−0.0007 (6)	0.0031 (6)
C9	0.0292 (9)	0.0364 (10)	0.0149 (8)	0.0076 (8)	−0.0005 (7)	0.0061 (7)
C10	0.0288 (10)	0.0413 (11)	0.0226 (9)	0.0061 (8)	0.0093 (7)	0.0128 (8)
C11	0.0205 (8)	0.0318 (10)	0.0206 (8)	0.0056 (7)	0.0066 (6)	0.0043 (7)
C12	0.0149 (7)	0.0213 (8)	0.0172 (7)	0.0024 (6)	0.0011 (6)	0.0012 (6)
C13	0.0159 (7)	0.0174 (7)	0.0137 (7)	0.0046 (6)	0.0003 (5)	0.0042 (6)
C14	0.0280 (9)	0.0168 (8)	0.0201 (8)	−0.0012 (6)	−0.0030 (7)	0.0042 (6)
C15	0.0375 (11)	0.0205 (9)	0.0269 (9)	−0.0007 (7)	−0.0027 (8)	0.0085 (7)
C16	0.0349 (10)	0.0301 (10)	0.0234 (9)	−0.0007 (8)	−0.0019 (7)	0.0134 (8)
C17	0.0305 (10)	0.0287 (10)	0.0203 (8)	−0.0006 (7)	−0.0073 (7)	0.0085 (7)
C18	0.0214 (8)	0.0192 (8)	0.0154 (7)	0.0000 (6)	−0.0035 (6)	0.0030 (6)
C19	0.0157 (7)	0.0135 (7)	0.0135 (7)	0.0012 (5)	0.0011 (5)	0.0008 (5)
C20	0.0147 (7)	0.0187 (8)	0.0219 (8)	0.0030 (6)	0.0006 (6)	0.0048 (6)
C21	0.0195 (8)	0.0216 (8)	0.0246 (9)	−0.0016 (6)	0.0022 (6)	0.0030 (7)
C22	0.0268 (9)	0.0202 (8)	0.0194 (8)	−0.0009 (7)	0.0055 (7)	0.0043 (6)
C23	0.0299 (9)	0.0182 (8)	0.0186 (8)	0.0051 (7)	0.0030 (7)	0.0063 (6)
C24	0.0179 (8)	0.0190 (8)	0.0177 (7)	0.0042 (6)	0.0008 (6)	0.0056 (6)
C25	0.0165 (7)	0.0193 (8)	0.0203 (8)	−0.0006 (6)	0.0006 (6)	0.0027 (6)
C26	0.0164 (7)	0.0151 (7)	0.0174 (7)	0.0019 (6)	−0.0011 (6)	0.0024 (6)
C27	0.0119 (7)	0.0136 (7)	0.0126 (7)	0.0031 (5)	−0.0010 (5)	−0.0013 (5)
C28	0.0176 (7)	0.0149 (7)	0.0120 (7)	0.0048 (6)	−0.0001 (5)	0.0002 (5)
C29	0.0171 (7)	0.0132 (7)	0.0141 (7)	0.0035 (5)	0.0005 (5)	0.0004 (5)
C30	0.0131 (7)	0.0153 (7)	0.0161 (7)	0.0002 (5)	0.0025 (5)	−0.0008 (6)
C31	0.0615 (17)	0.0422 (14)	0.0572 (16)	−0.0106 (12)	−0.0016 (13)	0.0174 (12)
C32	0.0617 (17)	0.0301 (12)	0.0684 (18)	−0.0043 (11)	−0.0048 (14)	0.0201 (12)
O8A	0.0669 (17)	0.0281 (12)	0.0749 (19)	0.0023 (11)	0.0341 (14)	0.0115 (11)
O8B	0.052 (5)	0.048 (5)	0.041 (4)	−0.006 (4)	−0.001 (3)	−0.001 (3)

### Geometric parameters (Å, º)

**Table:**

O1—C25	1.213 (2)	C14—C15	1.532 (3)
O2—C25	1.322 (2)	C14—H14A	0.9900
O2—H2	0.94 (3)	C14—H14B	0.9900
O3—C28	1.261 (2)	C15—C16	1.523 (3)
O4—C28	1.244 (2)	C15—H15A	0.9900
O5—C30	1.262 (2)	C15—H15B	0.9900
O6—C30	1.252 (2)	C16—C17	1.525 (3)
O7—C27	1.4273 (18)	C16—H16A	0.9900
O7—H5	0.86 (3)	C16—H16B	0.9900
N1—C7	1.502 (2)	C17—C18	1.535 (2)
N1—C1	1.502 (2)	C17—H17A	0.9900
N1—H1A	0.93 (2)	C17—H17B	0.9900
N1—H1B	0.87 (2)	C18—H18A	0.9900
N2—C19	1.510 (2)	C18—H18B	0.9900
N2—C13	1.512 (2)	C19—C20	1.523 (2)
N2—H2C	0.90 (2)	C19—C24	1.530 (2)
N2—H2D	0.93 (2)	C19—H19	1.0000
C1—C6	1.526 (2)	C20—C21	1.531 (2)
C1—C2	1.527 (2)	C20—H20A	0.9900
C1—H1	1.0000	C20—H20B	0.9900
C2—C3	1.532 (2)	C21—C22	1.529 (3)
C2—H2A	0.9900	C21—H21A	0.9900
C2—H2B	0.9900	C21—H21B	0.9900
C3—C4	1.525 (3)	C22—C23	1.530 (3)
C3—H3A	0.9900	C22—H22A	0.9900
C3—H3B	0.9900	C22—H22B	0.9900
C4—C5	1.526 (3)	C23—C24	1.531 (2)
C4—H4A	0.9900	C23—H23A	0.9900
C4—H4B	0.9900	C23—H23B	0.9900
C5—C6	1.536 (2)	C24—H24A	0.9900
C5—H5A	0.9900	C24—H24B	0.9900
C5—H5B	0.9900	C25—C26	1.521 (2)
C6—H6A	0.9900	C26—C27	1.547 (2)
C6—H6B	0.9900	C26—H26A	0.9900
C7—C8	1.523 (2)	C26—H26B	0.9900
C7—C12	1.528 (2)	C27—C29	1.543 (2)
C7—H7	1.0000	C27—C28	1.547 (2)
C8—C9	1.526 (2)	C29—C30	1.536 (2)
C8—H8B	0.9900	C29—H29A	0.9900
C8—H8C	0.9900	C29—H29B	0.9900
C9—C10	1.528 (3)	C31—C32	1.465 (4)
C9—H9A	0.9900	C31—H31A	0.9800
C9—H9B	0.9900	C31—H31B	0.9800
C10—C11	1.524 (3)	C31—H31C	0.9800
C10—H10A	0.9900	C32—O8B	1.243 (8)
C10—H10B	0.9900	C32—O8A	1.369 (4)
C11—C12	1.532 (2)	C32—H32A	0.9900
C11—H11A	0.9900	C32—H32B	0.9900
C11—H11B	0.9900	C32—H32C	0.9794
C12—H12A	0.9900	C32—H32D	0.9806
C12—H12B	0.9900	O8A—H32D	0.6660
C13—C18	1.520 (2)	O8A—H8AA	0.89 (5)
C13—C14	1.528 (2)	O8B—H8BB	0.89 (2)
C13—H13	1.0000		
			
C25—O2—H2	108.3 (19)	C17—C16—H16B	109.5
C27—O7—H5	104.4 (16)	H16A—C16—H16B	108.1
C7—N1—C1	117.54 (12)	C16—C17—C18	111.90 (15)
C7—N1—H1A	109.4 (14)	C16—C17—H17A	109.2
C1—N1—H1A	107.4 (13)	C18—C17—H17A	109.2
C7—N1—H1B	110.1 (15)	C16—C17—H17B	109.2
C1—N1—H1B	108.7 (15)	C18—C17—H17B	109.2
H1A—N1—H1B	102.7 (19)	H17A—C17—H17B	107.9
C19—N2—C13	118.47 (12)	C13—C18—C17	109.69 (14)
C19—N2—H2C	107.1 (14)	C13—C18—H18A	109.7
C13—N2—H2C	105.4 (13)	C17—C18—H18A	109.7
C19—N2—H2D	107.2 (14)	C13—C18—H18B	109.7
C13—N2—H2D	107.1 (14)	C17—C18—H18B	109.7
H2C—N2—H2D	111.5 (19)	H18A—C18—H18B	108.2
N1—C1—C6	112.10 (13)	N2—C19—C20	111.40 (13)
N1—C1—C2	107.64 (13)	N2—C19—C24	107.24 (13)
C6—C1—C2	111.36 (14)	C20—C19—C24	111.22 (13)
N1—C1—H1	108.5	N2—C19—H19	109.0
C6—C1—H1	108.5	C20—C19—H19	109.0
C2—C1—H1	108.5	C24—C19—H19	109.0
C1—C2—C3	110.90 (14)	C19—C20—C21	109.94 (14)
C1—C2—H2A	109.5	C19—C20—H20A	109.7
C3—C2—H2A	109.5	C21—C20—H20A	109.7
C1—C2—H2B	109.5	C19—C20—H20B	109.7
C3—C2—H2B	109.5	C21—C20—H20B	109.7
H2A—C2—H2B	108.0	H20A—C20—H20B	108.2
C4—C3—C2	111.19 (15)	C22—C21—C20	111.01 (14)
C4—C3—H3A	109.4	C22—C21—H21A	109.4
C2—C3—H3A	109.4	C20—C21—H21A	109.4
C4—C3—H3B	109.4	C22—C21—H21B	109.4
C2—C3—H3B	109.4	C20—C21—H21B	109.4
H3A—C3—H3B	108.0	H21A—C21—H21B	108.0
C3—C4—C5	110.55 (16)	C21—C22—C23	110.24 (14)
C3—C4—H4A	109.5	C21—C22—H22A	109.6
C5—C4—H4A	109.5	C23—C22—H22A	109.6
C3—C4—H4B	109.5	C21—C22—H22B	109.6
C5—C4—H4B	109.5	C23—C22—H22B	109.6
H4A—C4—H4B	108.1	H22A—C22—H22B	108.1
C4—C5—C6	111.25 (15)	C22—C23—C24	111.66 (14)
C4—C5—H5A	109.4	C22—C23—H23A	109.3
C6—C5—H5A	109.4	C24—C23—H23A	109.3
C4—C5—H5B	109.4	C22—C23—H23B	109.3
C6—C5—H5B	109.4	C24—C23—H23B	109.3
H5A—C5—H5B	108.0	H23A—C23—H23B	107.9
C1—C6—C5	109.55 (14)	C19—C24—C23	111.84 (14)
C1—C6—H6A	109.8	C19—C24—H24A	109.2
C5—C6—H6A	109.8	C23—C24—H24A	109.2
C1—C6—H6B	109.8	C19—C24—H24B	109.2
C5—C6—H6B	109.8	C23—C24—H24B	109.2
H6A—C6—H6B	108.2	H24A—C24—H24B	107.9
N1—C7—C8	107.98 (13)	O1—C25—O2	121.43 (16)
N1—C7—C12	112.60 (13)	O1—C25—C26	121.54 (15)
C8—C7—C12	110.47 (14)	O2—C25—C26	117.04 (15)
N1—C7—H7	108.6	C25—C26—C27	116.61 (13)
C8—C7—H7	108.6	C25—C26—H26A	108.1
C12—C7—H7	108.6	C27—C26—H26A	108.1
C7—C8—C9	110.04 (14)	C25—C26—H26B	108.1
C7—C8—H8B	109.7	C27—C26—H26B	108.1
C9—C8—H8B	109.7	H26A—C26—H26B	107.3
C7—C8—H8C	109.7	O7—C27—C29	109.82 (13)
C9—C8—H8C	109.7	O7—C27—C28	106.75 (12)
H8B—C8—H8C	108.2	C29—C27—C28	108.06 (12)
C8—C9—C10	111.31 (15)	O7—C27—C26	108.57 (13)
C8—C9—H9A	109.4	C29—C27—C26	111.65 (13)
C10—C9—H9A	109.4	C28—C27—C26	111.88 (13)
C8—C9—H9B	109.4	O4—C28—O3	124.12 (15)
C10—C9—H9B	109.4	O4—C28—C27	118.05 (14)
H9A—C9—H9B	108.0	O3—C28—C27	117.80 (14)
C11—C10—C9	111.24 (16)	C30—C29—C27	114.95 (13)
C11—C10—H10A	109.4	C30—C29—H29A	108.5
C9—C10—H10A	109.4	C27—C29—H29A	108.5
C11—C10—H10B	109.4	C30—C29—H29B	108.5
C9—C10—H10B	109.4	C27—C29—H29B	108.5
H10A—C10—H10B	108.0	H29A—C29—H29B	107.5
C10—C11—C12	111.51 (15)	O6—C30—O5	125.15 (15)
C10—C11—H11A	109.3	O6—C30—C29	116.73 (14)
C12—C11—H11A	109.3	O5—C30—C29	118.06 (14)
C10—C11—H11B	109.3	C32—C31—H31A	109.5
C12—C11—H11B	109.3	C32—C31—H31B	109.5
H11A—C11—H11B	108.0	H31A—C31—H31B	109.5
C7—C12—C11	109.03 (14)	C32—C31—H31C	109.5
C7—C12—H12A	109.9	H31A—C31—H31C	109.5
C11—C12—H12A	109.9	H31B—C31—H31C	109.5
C7—C12—H12B	109.9	O8B—C32—O8A	108.5 (5)
C11—C12—H12B	109.9	O8B—C32—C31	132.9 (5)
H12A—C12—H12B	108.3	O8A—C32—C31	116.5 (3)
N2—C13—C18	112.31 (13)	O8B—C32—H32A	41.3
N2—C13—C14	107.71 (13)	O8A—C32—H32A	108.2
C18—C13—C14	110.85 (14)	C31—C32—H32A	108.2
N2—C13—H13	108.6	O8B—C32—H32B	68.1
C18—C13—H13	108.6	O8A—C32—H32B	108.2
C14—C13—H13	108.6	C31—C32—H32B	108.2
C13—C14—C15	109.80 (14)	H32A—C32—H32B	107.3
C13—C14—H14A	109.7	O8B—C32—H32C	97.0
C15—C14—H14A	109.7	O8A—C32—H32C	77.6
C13—C14—H14B	109.7	C31—C32—H32C	105.2
C15—C14—H14B	109.7	H32A—C32—H32C	138.2
H14A—C14—H14B	108.2	H32B—C32—H32C	36.9
C16—C15—C14	111.54 (16)	O8B—C32—H32D	107.9
C16—C15—H15A	109.3	O8A—C32—H32D	27.0
C14—C15—H15A	109.3	C31—C32—H32D	105.9
C16—C15—H15B	109.3	H32A—C32—H32D	89.9
C14—C15—H15B	109.3	H32B—C32—H32D	134.1
H15A—C15—H15B	108.0	H32C—C32—H32D	104.5
C15—C16—C17	110.67 (16)	C32—O8A—H32D	42.0
C15—C16—H16A	109.5	C32—O8A—H8AA	110 (3)
C17—C16—H16A	109.5	H32D—O8A—H8AA	122.2
C15—C16—H16B	109.5	C32—O8B—H8BB	123 (10)
			
C7—N1—C1—C6	55.53 (19)	C14—C13—C18—C17	−58.33 (18)
C7—N1—C1—C2	178.33 (13)	C16—C17—C18—C13	56.5 (2)
N1—C1—C2—C3	−179.63 (14)	C13—N2—C19—C20	−53.92 (17)
C6—C1—C2—C3	−56.38 (19)	C13—N2—C19—C24	−175.83 (13)
C1—C2—C3—C4	55.4 (2)	N2—C19—C20—C21	−176.37 (13)
C2—C3—C4—C5	−55.8 (2)	C24—C19—C20—C21	−56.79 (18)
C3—C4—C5—C6	57.3 (2)	C19—C20—C21—C22	58.96 (18)
N1—C1—C6—C5	177.73 (15)	C20—C21—C22—C23	−57.82 (19)
C2—C1—C6—C5	57.06 (19)	C21—C22—C23—C24	54.74 (19)
C4—C5—C6—C1	−57.6 (2)	N2—C19—C24—C23	176.47 (13)
C1—N1—C7—C8	172.29 (13)	C20—C19—C24—C23	54.44 (18)
C1—N1—C7—C12	50.06 (18)	C22—C23—C24—C19	−53.39 (19)
N1—C7—C8—C9	176.78 (14)	O1—C25—C26—C27	−125.72 (18)
C12—C7—C8—C9	−59.69 (18)	O2—C25—C26—C27	54.4 (2)
C7—C8—C9—C10	56.4 (2)	C25—C26—C27—O7	161.88 (13)
C8—C9—C10—C11	−54.1 (2)	C25—C26—C27—C29	40.67 (19)
C9—C10—C11—C12	54.9 (2)	C25—C26—C27—C28	−80.58 (17)
N1—C7—C12—C11	−179.41 (14)	O7—C27—C28—O4	−19.87 (19)
C8—C7—C12—C11	59.77 (18)	C29—C27—C28—O4	98.20 (16)
C10—C11—C12—C7	−57.4 (2)	C26—C27—C28—O4	−138.50 (15)
C19—N2—C13—C18	−58.92 (18)	O7—C27—C28—O3	162.01 (15)
C19—N2—C13—C14	178.74 (13)	C29—C27—C28—O3	−79.92 (18)
N2—C13—C14—C15	−178.05 (14)	C26—C27—C28—O3	43.4 (2)
C18—C13—C14—C15	58.71 (19)	O7—C27—C29—C30	−56.54 (17)
C13—C14—C15—C16	−56.9 (2)	C28—C27—C29—C30	−172.62 (13)
C14—C15—C16—C17	55.0 (2)	C26—C27—C29—C30	63.94 (17)
C15—C16—C17—C18	−54.9 (2)	C27—C29—C30—O6	−162.66 (14)
N2—C13—C18—C17	−178.87 (14)	C27—C29—C30—O5	20.0 (2)

### Hydrogen-bond geometry (Å, º)

**Table:**

*D*—H···*A*	*D*—H	H···*A*	*D*···*A*	*D*—H···*A*
N1—H1*A*···O4^i^	0.93 (2)	1.84 (2)	2.7651 (19)	170 (2)
N1—H1*B*···O7	0.87 (2)	2.31 (2)	3.033 (2)	140.8 (19)
N1—H1*B*···O4	0.87 (2)	2.07 (2)	2.8390 (19)	146 (2)
N2—H2*C*···O6^ii^	0.90 (2)	1.90 (2)	2.794 (2)	170.3 (19)
N2—H2*D*···O5	0.93 (2)	1.86 (2)	2.751 (2)	160 (2)
O2—H2···O3	0.94 (3)	1.56 (3)	2.499 (2)	174 (3)
O7—H5···O5	0.86 (3)	1.92 (3)	2.6678 (19)	145 (2)
C18—H18*A*···O2^iii^	0.99	2.51	3.405 (2)	150
C20—H20*A*···O1^iii^	0.99	2.47	3.457 (2)	172
C32—H32*A*···O8*A*^iv^	0.99	2.56	3.458 (4)	151

Symmetry codes: (i) −*x*+1, −*y*+1, −*z*+1; (ii) −*x*+1, −*y*+2, −*z*+2; (iii) *x*−1, *y*, *z*; (iv) −*x*+1, −*y*+1, −*z*.
